# Effects of wound infection on prognosis after laparoscopic abdominoperineal resection of rectal cancer

**DOI:** 10.3389/fonc.2022.1036241

**Published:** 2023-01-04

**Authors:** Wang Huang, Zheng-qiang Wei, Yu-hao Qiu, Gang Tang, Hao Sun

**Affiliations:** ^1^ Department of Gastrointestinal Surgery, Chongqing University Cancer Hospital, Chongqing, China; ^2^ Department of Gastrointestinal Surgery, The First Affiliated Hospital of Chongqing Medical University, Chongqing, China

**Keywords:** rectal cancer, wound infection, cancer recurrence, cancer metastasis, laparoscopic abdominoperineal resection (LAPR)

## Abstract

**Background:**

In two facilities in Chongqing, this research sought to retrospectively evaluate the effects of perineal wound infection on survival after laparoscopic abdominoperineal resection (LAPR) of rectal cancer.

**Methods:**

To obtain clinical information on patients who underwent LAPR between January 2013 and December 2021, we performed a multicenter cohort study. A total of 473 patients were enrolled: 314 in the non-infection group and 159 in the group with perineal infection. The general data, perioperative conditions, and tumor outcomes between groups were analyzed. The infection rates, recurrence rates, and survival rates of the two centers were compared.

**Results:**

The age, height, weight, body mass index (BMI), preoperative complications, preoperative treatment, and intraoperative conditions of patients in the LAPR infection group were not statistically different from those in the non-infection group. The percentage of men, typical postoperative hospital stay, length of initial postoperative therapy, and recurrence and metastasis rates were all considerably higher in the infection group than those in the non-infection group. Wound infection was an independent factor affecting tumor recurrence and metastasis after LAPR as well as an independent factor shortening patient survival time according to multivariate analysis. The incidence of wound infection, the rate of recurrence, and the rate of mortality did not vary significantly across sites.

**Conclusion:**

Wound infection after LAPR increases the mean postoperative hospital stay, prolongs the time to first postoperative treatment, and decreases the disease-free survival (DFS) and overall survival (OS). Therefore, decreasing the rate of LAPR wound infection is expected to shorten the postoperative hospital stay and prolong the patient DFS and OS. Patients with postoperative infection may require intensive adjuvant therapy.

## Introduction

Colorectal cancer (CRC) is one of the most prevalent malignant tumors. Nearly 90% of patients with CRC undergo tumor resection (1). The most frequent postoperative consequence of CRC is surgical site infection (SSI), including wound infection, anastomotic leakage, and abdominal infection, with an infection rate as high as 45% (2). SSI leads to long postoperative hospital stays and increases the use of postoperative antibiotics, reoperation rate, and psychological stress in patients; in addition, SSI can lead to increased health care costs (3–5). Moreover, SSI decreases disease-free survival (DFS) and overall survival (OS) (6, 7). Rectal anastomotic leakage has been linked to a higher risk of tumor recurrence and shorter OS according to a meta-analysis (8). The relationship between postoperative wound infection, an important component of SSI, and the prognosis of CRC has not yet been reported.

Importantly, 40% of patients with rectal cancer must undergo abdominoperineal resection (APR) (9) despite progress in surgical techniques and rectal cancer treatments. Compared with other surgical methods, APR has a higher wound infection rate. After wound infection, the prognosis time is long. Perineal wound infection, in severe cases, may show wound nonunion or chronic sinus formation, thus resulting in long-term chronic inflammation. Related research has shown that tumor incidence and growth are significantly influenced by inflammation. Rectal anastomotic leakage leads to an increase in the local recurrence rate of tumors after surgery, which may be caused mainly by long-term local chronic inflammatory stimulation. For patients with postoperative perineal incision infection, a contaminated incision and poor local blood supply to the wound may lead to long healing times and long-term inflammation at the site of the tumor resection. Whether this inflammatory state might also increase the local recurrence rate and decrease the DFS and OS of patients was unknown.

This study was aimed at investigating the relationships between perineal wound infection and tumor recurrence, metastasis, and survival after laparoscopic abdominoperineal resection (LAPR) to serve as a standard of comparison for the clinical diagnosis and management of rectal cancer.

## Patients and methods

### Clinical data

To incorporate the case data from the two sites in Chongqing, China, we conducted a retrospective cohort analysis. Retrospective data collection was conducted for patients with rectal cancer treated at the Chongqing University Cancer Hospital and the First Affiliated Hospital of Chongqing Medical University between January 2013 and December 2021. The inclusion criteria were as follows: 1) biopsy-confirmed adenocarcinoma of the rectum, 2) patient consent to LAPR, and 3) radical resection. The exclusion criteria were as follows: 1) history of other malignant tumors, multiple primary CRCs, or pathological diagnosis of non-adenocarcinoma; 2) anal preservation; 3) combined organ resection; 4) non-laparoscopic surgery or conversion to open administration; 5) history of radiotherapy for conditions other than rectal cancer; 6) no radical operation or clinical stage IV (including inguinal lymph node metastasis or lateral lymph node metastasis); and 7) unknown clinical information or loss to follow-up.

According to the above criteria, a total of 619 individuals with LAPR were identified, but 139 patients were excluded because of insufficient clinical information or loss to follow-up. Finally, 473 cases were included. Among them, 165 cases were enrolled at the Chongqing University Cancer Hospital, and 308 cases were enrolled at the First Affiliated Hospital of Chongqing Medical University.

According to the inclusion and exclusion criteria, participants were divided into a perineal incision infected group and a non-infected group according to the presence of perineal incision infection. All patients were operated on by experienced senior physicians.

### Preoperative therapeutic schedule

Every patient who was included underwent a thorough preoperative assessment, which included a pelvic MRI, colonoscopy, enhanced CT of the chest and abdomen, and tumor markers. Preoperative neoadjuvant chemoradiotherapy is recommended for patients with preoperative T stage T3 or T4, N stage N1 or N2, positive perioperative margin [circumferential resection margin (CRM)], or positive extramural vascular invasion (EMVI). The neoadjuvant chemoradiotherapy regimen comprised conventional long-term radiotherapy with a single dose of 1.8–2.0 Gy administered a total of 25–28 times. For 8–12 weeks of preoperative chemotherapy, the regimen included fluorouracil or capecitabine alone or a combination of CapeOX (capecitabine and oxaliplatin) or FOLFOX (fluorouracil and oxaliplatin). At 8–12 weeks after the end of radiotherapy, surgical treatment was performed after evaluation of the specific condition of the patient’s tumor.

### Operation

For abdominal surgery, the rectum was separated from the levator ani plane according to the total mesorectal excision (TME) principle, and the sigmoid colon was dissected 10 cm above the tumor. Extraperitoneal stoma or transrectus abdominis stoma were used for stoma. For perineal surgery, the patient was still in the lithotomy position. The anus was closed with a double purse-string suture, and the skin on both sides of the perineum and back and the adipose tissue of the ischial anal canal were dissected according to the standard APR scope. The adipose tissue was first separated from the sacrococcygeal region in the abdominal cavity, and then the adipose tissue of the ischial anal canal was gradually separated and incised from both sides. The posterior margin of the superficial transperineum muscle was incised in the front, and the anterior part of the rectum was connected to remove the specimen. After the wound was completely hemostatic, the pelvic and abdominal wounds were washed with warm water, the perineum was redisinfected and covered with towels, the presacral drainage tube and subcutaneous negative pressure drainage ball were indwelled, and the subcutaneous tissue and skin were sutured with a tension-reducing needle at intervals and full thickness.

### Postoperative adjuvant treatment

Pharmacy medication records were consulted, and patient in-hospital data or telephone follow-up data were collected. Postoperative adjuvant chemotherapy included fluorouracil or capecitabine alone, CapeOX, or FOLFOX.

### Follow-up

All patients underwent follow-up evaluations in the outpatient clinic 3–6 months postoperatively. Every 3 months, tests for tumor markers, including at least blood levels of carbohydrate antigen 19-9 (CA19-9) and carcinoembryonic antigen (CEA), were performed. Enhanced CT scans of the abdomen and pelvis were conducted once every 6 months, and a colonoscopy was performed once per year. Patients who did not return to the hospital for reexamination were followed up by telephone according to a schedule, and the survival status, symptoms of discomfort, and local examination results were recorded. Study follow-up continued until 1 July 2022.

### Observation indicators and evaluation criteria

General data, the perioperative period, and tumor prognosis between groups were analyzed. The infection rate, recurrence rate, and survival rate were compared between centers. This study mainly compared the prognosis of tumors between groups, including local recurrence and distant metastasis. Local recurrence refers to local tumors in the pelvic and perineal regions, as confirmed by imaging or reoperation pathology. The distant recurrence rate was defined as metastasis/recurrence of non-local recurrence sites, as confirmed by imaging or reoperation pathology.

### Statistical analysis

SPSS 23.0 software was used for statistical evaluation. Quantitative information was presented as Xs, and t-tests were used to compare groups. In this study, [n (%)] was used to express categorical data. For group comparison and univariate analysis, we used chi-square or Fisher exact test. In the analysis of the survival curve, multivariate logistic analysis was applied to characterize OS and DFS.

## Results

### Basic data analysis

The total infection incidence for perineal wounds was 33.62%; there were 159 instances of infection and 314 cases without infection. No significant differences were observed in age, BMI (weight/height^2^), comorbidities, and preoperative treatments between groups (P > 0.005, **Table 1**). The percentage of men in the experimental group was much greater than that in the control group (P < 0.005). The preoperative neoadjuvant therapy was long-term radiotherapy, followed by 6–12 weeks of neoadjuvant chemotherapy, followed by radical surgery.

**Table 1:** Patient characteristics.

	Infection group (n=159)	Non-infection group (n=314)	P
Gender Male Female	87 (54.72%) 72 (45.28%)	204 (64.96%) 110 (35.03%)	0.030
Age (years)	59.67±13.089	60.58 ± 10.937	0.452
Height (cm)	160.82 ± 9.238	161.47 ± 7.717	0.448
Weight (kg)	59.81 ± 10.935	59.19 ± 9.995	0.537
BMI (kg/m^2^)	23.07 ± 3.372	22.64 ± 3.051	0.159
History of smoking Yes No	37 (23.27%) 122 (76.73%)	83 (26.43%) 231 (73.57%)	0.455
History of alcohol consumption Yes No	40 (25.17%) 119 (74.85%)	75 (23.89%) 239 (76.11%)	0.761
Diabetes mellitus Normality Abnormality	14 (8.81%) 145 (91.19%)	25 (7.96%) 289 (92.04%)	0.753
Hypertension Normality Abnormality	33 (20.75%) 126 (79.24%)	46 (14.65%) 268 (85.35%)	0.093
Neoadjuvant therapy Yes No	24 (15.09%) 135 (84.91%)	41 (13.06%) 273 (86.94%)	0.543

### Operation and pathological stage

No significant differences were observed in the operation time, blood loss, distance between tumor and anus, tumor size, T stage, N stage, tumor stage, and number of positive lymph nodes between groups (P > 0.05, **Table 2**). The average length of hospital stay in the infection group was significantly longer than that in the non-infection group (P < 0.05). The distance was the shortest path between the tumor’s bottom margin and the anus. Tumor size referred to the longest tumor diameter. Six patients achieved a pathological complete response (PCR) after preoperative treatment.

**Table 2:** Surgical conditions and postoperative pathological features.

	Infection group (n=159)	Non-infection group (n=314)	P
Operating time (min)	265.28 ± 85.769	254.80 ± 77.805	0.182
Intraoperative bleeding (ml)	129.59 ± 112.902	142.52 ± 186.722	0.423
Distance (cm)	3.09 ± 1.499	3.18 ± 1.297	0.516
Tumor size (cm)	4.40 ± 1.902	4.13 ± 1.579	0.101
T stage T0 T1 T2 T3 T4	2 (1.26%) 8 (5.03%) 47 (29.56%) 55 (34.59%) 47 (29.56%)	4 (1.27%) 11 (3.50%) 81 (25.79%) 105 (33.44%) 113 (35.99%)	0.619
N stage N0 N1 N2	103 (64.78%) 29 (18.24%) 27 (16.98%)	191 (60.83%) 78 (24.84%) 45 (14.33%)	0.248
Pathological stage 0 I II III	2 (1.26%) 40 (25.16%) 59 (37.11%) 58 (36.48%)	4 (1.27%) 74 (23.57%) 120 (38.22%) 116 (36.94%)	0.974
Positive lymph node (n)	1.81 ± 3.735	1.39 ± 2.761	0.204
Differentiation degree Poorly Moderately Well	5 (3.14%) 150 (94.34%) 4 (2.52%)	15 (4.78%) 292 (92.99%) 7 (2.23%)	0.697
Postoperative hospital stay (days)	19.31 ± 14.148	12.62 ± 5.336	0.000

### Adjuvant therapy

Postoperative adjuvant medication was administered to 192 patients in the non-infection group and 102 patients in the infection group. The initial chemotherapy session lasted substantially longer in the experimental group than that in the control group (P < 0.05), whereas the number of postoperative adjuvant chemotherapy showed no difference (P > 0.05), as shown in **Table 3**.

**Table 3:** Time and frequency of postoperative chemotherapy.

	Infection group (n=102)	Non-infection group (n=192)	P
Time of first chemotherapy (days)	43.82 ± 16.337	29.91 ± 11.012	0.000
Frequency	5.11 ± 1.794	5.41 ± 1.682	0.368

### Follow-up

**Figure 1:**
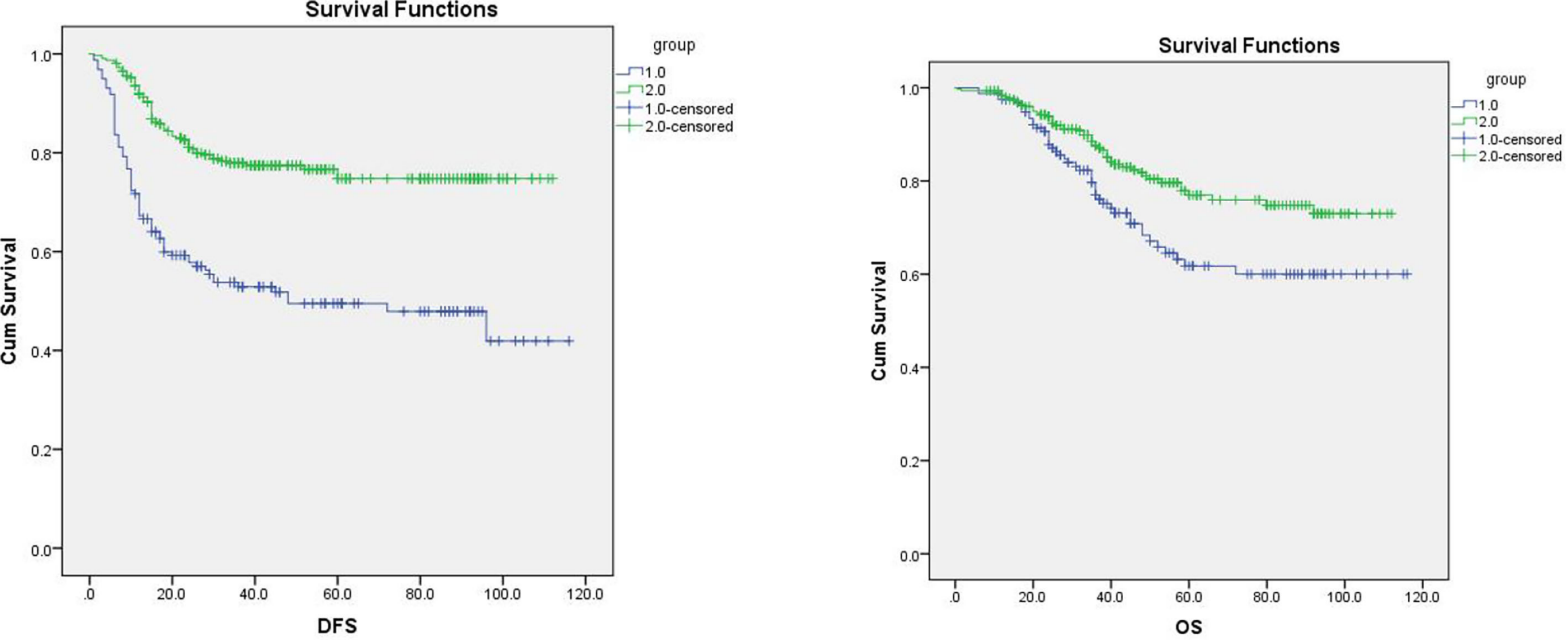
Overall survival (OS) and PFS of the two groups. Note: Group 1 is the infection group, and group 2 is the non-infection group. The longest follow-up was 120 months, in which changes in disease-free survival (DFS) and overall survival (OS) were significant (P < 0.05).

In the follow-up, in comparison to those in the non-infection group, the infection group’s rates of recurrence and metastasis, local recurrence, and death were all considerably higher (P < 0.005, **Table 4**). In the infection group, 77 cases had recurrence and metastasis, whereas in the non-infection group, 68 cases had recurrence and metastasis. In the first recurrence and metastasis, the local recurrence rate of the infected group was much higher than that of the non-infection group (77.92% vs. 48.53%). DFS (P = 0.000) and OS (P = 0.005) significantly decreased in the infection group (**Figure 1**).

**Table 4:** Prognosis.

	Infection group (n=159)	Non-infection group (n=314)	P
Recurrence/Metastasis Yes No	77 (48.43%) 82 (51.57%)	68 (21.66%) 246 (78.34%)	0.000
Position Local Local and distant Distant	37 (48.05%) 23 (29.87%) 17 (22.08%)	19 (27.94%) 14 (20.59%) 35 (51.47%)	0.001
Death Yes No	45 (28.30%) 114 (71.70%)	53 (16.88%) 261 (83.12%)	0.004

### Comparison between centers

The infection rate, postoperative average length of hospital stay, metastasis rate, and mortality rate did not significantly differ between centers (**Table 5**). The overall infection rate in the two centers was 42.63%, the recurrence rate of metastasis was 30.65%, and the mortality rate was 20.72%.

**Table 5:** Comparative analysis of the two centers.

	First Affiliated Hospital (n=308)	Cancer Hospital (n=165)	P
Infection Yes No	105 (34.09%) 203 (65.91%)	54 (32.73%) 111 (67.27%)	0.765
Postoperative hospital stay (days)	14.56 ± 11.126	15.44 ± 6.620	0.352
Recurrence/Metastasis Yes No	95 (30.84%) 213 (69.16%)	50 (30.30%) 115 (69.70%)	0.903
Death Yes No	70 (22.73%) 238 (77.27%)	28 (16.97%) 137 (83.03%)	0.141

### Multiple-factor analysis

Univariate analysis of postoperative metastasis and recurrence of rectal cancer indicated that body weight, BMI, operation time, number of positive lymph nodes, N stage, tumor stage, infection, and postoperative hospital stay were statistically significant. After adjustment for the above factors, the risk of recurrence and metastasis was increased in patients with vaginal wound infection (odds ratio (OR) = 3.526, 95% CI: 2.228–5.578, P = 0.000). Univariate analysis of death due to rectal cancer indicated that the operation time, number of positive lymph nodes, N stage, tumor stage, infection, and postoperative hospital stay were statistically significant. After adjustment for the above factors in the logistic regression model, the perineal wound infection group had an increased risk of death (OR = 1.815, 95% CI: 1.107–2.976, P = 0.018).

## Discussion

CRC has a high incidence and mortality. In 2020, globally, more than 1.9 million new cases of CRC and 935,000 deaths have been estimated to result from CRC, accounting for approximately one-tenth of all cancer cases and fatalities (10). However, the incidence and mortality of CRC are almost twice as high in men than those in women (9). In the data included in this study, the incidence was approximately 1.60 times higher in men than that in women, in line with the tumor distribution. The overall death rate for individuals with rectal cancer in this study was 20.72%, a finding consistent with the high mortality rate reported in the literature.

CRC is treated mainly with surgery. In China, the incidence of rectal cancer accounts for approximately 50% of CRCs, whereas lower rectal cancer accounts for 60%–70% of all CRCs. Postoperative complications of rectal cancer are significantly higher than those of colon cancer (11), with rates reaching 40% (12), according to the literature. However, APR has higher postoperative complications; most data have indicated an incidence of perineal complications of 10.1%–45% (9, 13–15). After preoperative neoadjuvant chemoradiotherapy, the incidence of perineal complications can even reach 60%–70% (9, 16). Wound infection is the main complication in the perineal area after APR. The perineal wound infection rate was found to be 32.73%, in agreement with the literature, possibly because the sacral cavity forms a large wound area after rectum resection, thus resulting in fluid accumulation and pelvic abscess. In addition, the operation time of LAPR is longer, thus potentially increasing the risk of postoperative infection. Wound infection increases medical expenses, prolongs hospital stay, and decreases patient quality of life (17). In this study, in comparison to that in the non-infection group, the average postoperative hospital stay in the infected group was much longer (19.31 days vs. 12.62 days).

After CRC surgery, SSI can decrease the DFS after radical surgery (6, 7) but has not been demonstrated to be associated with OS. Anastomotic leakage after rectal surgery promotes local recurrence and decreases DFS and OS according to several studies (18–22). However, anastomotic leakage has been found to increase local recurrence without affecting OS or DFS (23). Thus, this conclusion is controversial at present. In gastric cancer, SSI has been reported to decrease OS after radical surgery (24), and anastomotic leakage has been found to decrease OS after gastric cancer surgery according to several studies (25, 26). However, this conclusion is still debatable. Anastomotic leakage after treatment for stomach cancer, according to some research, has no effect on prognosis (27). However, no study has examined the relationship between wound infection and prognosis after gastric CRC surgery. This study showed that perineal wound infection increased the local tumor recurrence rate and decreased the OS and DFS. Simultaneously, after rectal cancer surgery, perineal wound infection is a separate risk factor for both DFS and OS.

At present, the mechanism of LAPR wound infection and poor tumor prognosis is unclear. Inflammation may be activated by wound infection in the perineal region. However, inflammatory cells produce tumor necrosis factor-α, transforming growth factor-β, interleukin-6 (IL-6), and other cytokines, which regulate the transcription factor NF-κB and the signal transducer and activator of transcription-3 (STAT3) pathways, and promote tumor cell metastasis (28–30). Another research has shown that inflammatory cells cause overexpression of vascular endothelial growth factor (VEGF) and IL-6 (31). The most potent angiogenic cytokine is VEGF, and angiogenesis plays a major role in tumor spread and recurrence (32). Shorter DFS and OS are associated with elevated blood VEGF levels in patients with CRC (33, 34). In this study, wound infection in the perineal area of LAPR resulted in the activation of inflammatory cells, which might have led to the systemic inflammatory response syndrome, thereby increasing the risk of postoperative tumor spread. Furthermore, in CRC, after resection, cancer cells that are still present in the large intestine’s mucosa and intestinal lumen may peel off and become implanted in the surrounding area (35). In addition, inflammation in the abdomen can help cancer cells adhere together, move around, and invade other tissues, whereas the wound infection in the perineal area after LAPR is mainly confined to the pelvic cavity, thus resulting in local adhesion, tumor cell invasion, and migration. Consequently, in comparison to the non-infection group, the infection group’s local recurrence rate was significantly greater (77.92% vs. 48.53%). Finally, postoperative adjuvant chemotherapy may prolong OS and decrease postoperative recurrence in stage II/III rectal cancer (36). According to National Comprehensive Cancer Network (NCCN) guidelines, postoperative adjuvant chemotherapy should be performed within 3 weeks and generally not more than 8 weeks. According to a meta-analysis, extended adjuvant chemotherapy beyond 8 weeks dramatically shortens DFS and OS (37, 38). In our study, although the mean time to the first postoperative chemotherapy in the infected group did not exceed 8 weeks, it was much longer than that in the non-infection group. This finding might indicate one factor contributing to the infected group’s elevated risk of local recurrence.

Studies have shown that minimally invasive techniques can reduce SSI (39); this conclusion has also been confirmed in CRC (40, 41) and through urology (42). Moreover, LAPR combined with pelvic peritoneal closure can decrease the infection rate after APR (43). The postoperative infection incidence of rectal cancer may be decreased by oral antibiotics and mechanical bowel preparation (44). Preoperative neoadjuvant chemoradiotherapy, the main treatment for locally advanced rectal cancer, has been found to decrease the tumor stage and thus improve the R0 removal rate of tumors, and this conclusion has been confirmed with total neoadjuvant therapy (TNT) (45–47). However, neoadjuvant chemoradiotherapy has been the most frequently documented risk factor for SSI after APR in recent years (48–50). Unfortunately, no available evidence suggests that preoperative neoadjuvant treatment increases the incidence of LAPR wound infection. In this study, only 13.74% of patients received preoperative radiotherapy. Therefore, in LAPR, preoperative mechanical bowel preparation, oral antibiotics, intraoperative aseptic procedures, and closure of the basin peritoneum may limit the wound infection rate and thus improve tumor prognosis.

This study’s primary limitation was its retrospective methodology. However, we collected data continuously from two institutional databases to avoid data selection bias to some extent. However, this conclusion still must be confirmed in a prospective multicenter large-sample study.

## Conclusions

Wound infection after LAPR increased the postoperative hospital stay, delayed the time of postoperative first adjuvant chemotherapy, increased the postoperative tumor recurrence and metastasis, and decreased the survival time in patients. Therefore, limiting the wound infection rate of LAPR is expected to shorten the postoperative hospital stay, decrease the time of the first adjuvant chemotherapy, and improve the DFS, OS, and tumor prognosis. Intensive postoperative adjuvant therapy may be needed in patients with postoperative infection.

## Data availability statement

The raw data supporting the conclusions of this article will be made available by the authors, without undue reservation.

## Author contributions

WH made important contributions to the research conception and design, as well as to the analysis and interpretation. WH, Y-hQ, and GT participated in the data collection. WH wrote the manuscript. HS and Z-qW supervised and edited the manuscript. WH is the first author, and HS is the corresponding author. All authors contributed to the article and approved the submitted version.
